# Foliar Application of Bio-Stimulants Enhancing the Production and the Toxicity of *Origanum majorana* Essential Oils Against Four Rice Seed-Borne Fungi

**DOI:** 10.3390/molecules25102363

**Published:** 2020-05-19

**Authors:** Abeer A. Mohamed, Mervat El-Hefny, Nader A. El-Shanhorey, Hayssam M. Ali

**Affiliations:** 1 Plant Pathology Institute, Agriculture Research Center (ARC), Alexandria 21616, Egypt; abeer_pcr@yahoo.com; 2 Department of Floriculture, Ornamental Horticulture and Garden Design, Faculty of Agriculture (El-Shatby), Alexandria University, Alexandria 21545, Egypt; 3 Department of Botanical Gardens Research, Horticultural Research Institute (ARC), Alexandria 21554, Egypt; dr_shanhorey@yahoo.com; 4 Botany and Microbiology Department, College of Science, King Saud University, P.O. Box 2455, Riyadh 11451, Saudi Arabia; hayhassan@ksu.edu.sa; 5 Timber Trees Research Department, Sabahia Horticulture Research Station, Horticulture Research Institute, Agriculture Research Center, Alexandria 21526, Egypt

**Keywords:** *Origanum majorana* plants, ascorbic acid, tryptophan, moringa leaf extract, essential oils, rice, antifungal activity, grain discoloration disease

## Abstract

In the present study, the enhancement of the production of *Origanum majorana* essential oils (EOs) was studied by treating plants with ascorbic acid (AA) and tryptophan (Trp) at concentrations of 100, 200 and 300 mg/L and *Moringa oleifera* leaf extract (MLE) at 2.5%, 5% and 10% as foliar applications during the seasons 2018–2019. The toxicities of the EOs were assayed against four seed-borne fungi (*Bipolaris orzyae*, *Curvularia lunata*, *Fusarium verticilliodies* and *F. graminearum*) isolated from rice grains (*Oryzae sativa*). Vegetative growth parameters and EO production were enhanced by the application of AA, Trp and MLE in both seasons. Analysis of the EOs by Gas chromatography–mass spectrometry (GC-MS) showed that the main chemical constituents were terpineol (*cis*-*β*-(1-terpinenol)), terpinen-4-ol, 4-thujanol (sabinene hydrate), *α*-terpineol, cymene and sabinene. The highest fungal mycelial growth inhibition (FMGI) percentages against *F. verticilliodies* were 94.57% and 92.63% as MLE at 5% and 10%, respectively, was applied to plants and 85.60% and 82.19% against *F. graminearum* as Trp was applied to plants at 300 and 200 mg/L, respectively. EOs from the treated plant with MLE (10%) observed the highest FMGI (84.46%) against *B. oryzae*, and EOs from plants treated with AA as foliar application at 300 and 200 mg/L showed the highest FMGI values of 81.11% and 81.85%, respectively, against the growth of *C. lunata*. Application of EOs extracted from plants treated with Trp, AA and MLE at 300 mg/L, 300 mg/L and 10%, respectively, or untreated plants to rice seeds inhibited or decreased the fungal infection percentage from 82.5% (naturally infected grains) to 1.75%, 10.5%, 17.5% and 18.5%, respectively. In conclusion, the extracted EOs affected by the foliar application of *O. majorana* plants with Trp, AA, and MLE could be useful as a biofungicide against rice seed-borne fungi.

## 1. Introduction

*Origanum majorana* L. (marjoram) belongs to the family Lamiaceae, is a perennial aromatic herb native to Cyprus in the Mediterranean region and is widely distributed in Egypt. It is one of the most important medicinal and aromatic plants and is considered as an important economic agricultural export crop [1,2]. Marjoram, a herb, and its essential oils (EOs), are used in the food industry in condiments and as food preservatives, and it is also used for its medicinal properties as a stomachic, carminative, expectorant and antispasmodic agent [3,4,5]. Marjoram EO has been studied for its antibacterial, antifungal and antiviral activities, as an insecticide and in pharmaceutical and industrial products [6,7,8,9,10]. These activities could be related to its main compounds including terpinene-4-ol, *cis*-sabinene hydrate, *α*- and *γ*-terpinene, *α*-phyllandrene and carvacrol [6,7,9].

There are several kinds of bio-stimulants including micronutrients, proteins, amino acids, enzymes, phenols, fulvic acids and salicylic acid [11,12]. Natural extracts can be used as bio-stimulants for both agricultural and horticultural crops to improve the growth, general health and vitality of plants, as well as to protect them against infections [13]. It was proven to improve crop quality and increase nutrient utilization efficiency and plant tolerance to abiotic stress [13,14,15]. Natural extracts are a good source of bio-stimulants. In one example, rosemary extract at 1000 mg/L was reported to stimulate the growth of tomato plants, while nutrient uptake and the fresh mass of roots were improved by the application of the EO [16]. Foliar application of bio-stimulants is more effective than soil application. In one study, the levels of macro- and micronutrients increased in maize leaves as the foliar application of sewage sludge bio-stimulant was applied [17].

*Moringa oleifera* Lam. belongs to the family Moringaceae and is a tropical crop grown for its nutritional and medicinal purposes [18,19]. It can be used as a bio-stimulant for plants, where its leaves are considered as a source of some vitamins (A, B, C), minerals (K, Ca, Fe) and amino acids [20,21]. Natural antioxidants such as ascorbate and phenolic components have been identified in moringa leaf extracts (MLE) [22]. Moreover, moringa leaf extracts (MLEs) are rich in cytokinins, auxins, abscisic acid, zeatin, ascorbates, vitamin E, phenolics and minerals [18,23].

Foliar spray application of crops with MLE has increased plant growth and yield and improved the resistance of plants to stresses [18]. Moreover, MLE acts as a plant bio-stimulant when applied alone in seed soaking and/or when applied as foliar spray leading to modified plant growth and production with positive alterations on metabolic processes under salt stress conditions [24,25]. MLE sprayed onto leaves of melon, onions, bell pepper, soya beans and chili led to an increase in yield [24].

Vitamins and amino acids have been proven to play important roles in promoting the productivity of plants. For example, ascorbic acid (AA) plays an important role in several physiological processes such as regulation of enzymes and controlling cell division and expansion, protein modification, growth, development and senescence [26,27,28,29]. Additionally, tryptophan (Trp) has an indirect role on growth via auxin synthesis [30]. Several alternative roles of indole acetic acid (IAA) synthesis have been suggested in plants, all starting from Trp; thus, when Trp is supplied to most plant tissues, IAA is formed [30]. Furthermore, foliar application of amino acids (Trp) enhanced the vegetative growth and chemical constituents of basil plants [31] and *Pelargonium graveolens* L. and *Catharanthus roseus* L. [32].

Cultivated crops can be infected by many fungal pathogens causing several diseases which lead to economic losses. The majority of these diseases are caused by seed-borne fungi. Phytometabolites and plant extract pesticides are some of the better alternatives to control fungal diseases; they have minimal environmental problems and pose less danger to consumers in contrast to synthetic pesticides [33,34,35]. Subsequently, in this study, the EOs obtained from *O. majorana* plants affected by the addition of MLE, AA, and Trp in foliar applications were used as an antifungal agent against rice seed-born fungi.

Rice (*Oryzae sativa* L.) is one of the most important cereal crops cultivated in Egypt and in tropical countries of the world, and feeds more than half of the world’s population [36]. Grain discoloration of rice is a complex disease due to infection by certain microorganisms on the glumes, kernels or both. The fungi that are reported to be associated with discoloration of grains are *Pyricularia oryzae*, *Fusarium verticilliodies* (bakana), *F. graminearum* (head blight), *Nigrospora oryzae*, *Epicoccum nigrum*, *Curvularia lunata* (leaf spot) and *Bipolaris orzyae* (brown spot) [37,38].

Therefore, the aims of this study were, firstly, to evaluate the effects of AA, Trp and MLE follicular application on the vegetative growth, chemical parameters and oil production (yield and components) of *O. majorana* plants and, secondly, to investigate the antifungal activity of the essential oils against major seed-born rice fungi.

## 2. Results and Discussion

### 2.1. Field Study

#### 2.1.1. Effects of Ascorbic Acid, Tryptophan and Moringa Leaf Extract on Vegetative Growth

Results in Table 1 show the significant effects of different concentrations of ascorbic acid (AA), tryptophan (Trp) and moringa leaf extract (MLE) on the vegetative parameters of *O. majorana* plants (plant height (cm), plant diameter (cm), branch number/plant, leaf fresh weight (g), leaf dry weight (g) and leaf area (cm^2^)) compared with control plants in both seasons (2018–2019). The highest value of growth was obtained by the plants treated with AA at a concentration of 300 mg/L. These results agree with Hashem [2] who found that foliar spraying of marjoram plants with AA at concentrations of 150 and 300 mg/L significantly increased all vegetative parameters compared to control plants. Another study showed that application of MLE at 75 mL/L positively increased the vegetative growth of *Pelargonium graveolens* L’Hér. [39]. Furthermore, at high rates MLE significantly increased the fresh and dry weights of tomato leaves [40]. Foliar application of AA increased the plant growth yield of peas (*Pisum sativum* L.,) roselle (*Hibiscus sabdariffa* L.) and fennel (*Foeniculum vulgare* Mill.) plants [41,42,43]. Application of AA at different concentrations showed significant increases in all growth parameters and fresh and dry weights of *Ocimum basilicum* L. plant [44].

The simulative effect of MLE on herb growth due to its beneficial effects and supplying the growing plants with the required micro and macronutrient elements plays an important role in metabolic processes such as photosynthesis and carbohydrate synthesis [39]. The simulative effects of AA and Trp on herb growth resulted from their important roles in controlling several physiological processes and synthesis of plant hormones [32]. Furthermore, Trp plays a direct role in regulating plant development and pathogen defense responses [45], which promote the growth of plants. Through eliciting the synthesis of endogenous phytohormones such as auxin and gibberellin, bio-stimulant applications are responsible for activating the transduction signal pathway, which subsequently leads to a higher yield [46,47].

#### 2.1.2. Effects of Ascorbic Acid, Tryptophan and Moringa Extract on the Chemical Parameters of *O. majorana*

##### Oil Percentage (%)

There was a significant effect of the different treatments on the essential oil (EO) percentage of *O. majorana*, where the highest percentage was obtained by plants treated with MLE (10%) in both seasons (2018–2019), with values of 1.06% and 0.86%, respectively, compared to control treatments (0.66% and 0.65%; Table 2). These results are in line with those obtained by Abd-el-Kader and Hamad [42], where they noticed that Roselle plants treated with 300 mg/L AA had improved vegetative parameters, yield production and chemical constituents of Roselle sepals. Sakr et al. [39] indicated that foliar applications of MLE significantly improved the EO percentage and yield per plant and feddan of geranium plants. Moreover, application of AA increased the EO yield of *O. basilicum* plant [43].

##### Total Chlorophyll and Carbohydrate Content

There were significant effects of the studied treatments on total chlorophyll and total carbohydrate contents of *O. majorana* plants compared with the control plants in both seasons (Table 2). The best results were obtained by plants treated with the highest concentration of MLE (10%). These results agreed with Sakr et al. [39] who reported that foliar application to geranium plants with MLE at 6 g/L increased the values of chlorophyll a, chlorophyll b and carotenoids. This increase may be due to moringa leaves having a high content of different macro elements such as Mg, a constituent of chlorophyll that would be responsible for inducing chlorophyll (a and b) in leaves with increasing carbohydrate content [48].

##### Chemical Composition of the Essential Oils

Table 3 lists the chemical compounds of *O. majorana* leaf EOs as analyzed by Gas chromatography–mass spectrometry (GC-MS).

These compounds were affected by different foliar applications applied to the plants compared with the control. Terpineol ranged from 38.06% (AA at 300 mg/L) to 26.76% (AA at 100 mg/L); terpinen-4-ol ranged between 38.35% and 27.65%, where the highest value was obtained by Trp at 300 mg/L; and the percentage of 4-thujanol (sabinene hydrate) ranged from 46.47% (MLE 2.5%) to 9.13%. The cymene percentage ranged from 17.75% to 4.5%, where the maximum relative percentage was obtained by Trp at 300 mg/L. *α*-Terpineol was found in the range of 12.58% to 6.24%, where the highest percentage was obtained by MLE at 10%, and sabinene ranged from 8.66% to 2.76%. In addition, the oxygenated constituents of EOs ranged from 62.88% to 89.17% and non-oxygenated constituents of EOs ranged from 9.62% to 26.65%. These ranges show strong positive correlation coefficients among the treatments in terms of affecting the oxygenated and non-oxygenated percentages as shown in Table 4.

The results of the current investigation agree with other observations [2,49]. There were two different chemotypes of *O. majorana* L. The oil of the first chemotype was rich in monoterpene alcohols including terpinen-4-ol (10%–48%), *cis*-sabinene hydrate (0.2%–33.0%), *trans*-sabinene hydrate and *α*-terpineol. Terpinen-4-ol is the substance responsible for the odor of these oils. The second chemotype included the phenolics thymol (0.01%–21.1%) and carvacrol (0.4%–30.0%) as major constituents [50,51]. In addition, these results are in line with Elansary [52] and Gharib et al. [53], who found that the main EO constituents of marjoram growing in Northwest Egypt were *cis*-sabinene hydrate, *α*-terpinene, 4-terpineol and sabinene. These effects on EO production may be due to the foliar application of AA, which is an essential co-factor for α-ketoglutarate-dependent dioxygenases (e.g., prolyl hydroxylases), which play an important role in the formation of covalent adducts with electrophilic secondary metabolites in plants [54]. Furthermore, it is responsible for several metabolic enzymes involved in the fundamental developmental process of plants [55]. Additionally, aromatic amino acids—phenylalanine, tyrosine and Trp—are central molecules in plant metabolism. Besides their function in building proteins, they serve as precursors for a variety of plant hormones, such as auxin and salicylate, as well as for a very wide range of aromatic secondary metabolites [56,57].

### 2.2. In Vitro Study

#### 2.2.1. Isolation and Identification of the Fungal Isolates

The identifed *Curvularia lunata* fungal isolate from rice grains of Giza 177 cultivar showed grain discoloration. Three fungal isolates, *Fusarium verticilliodies* (F.v.101), *F. graminearum* (F.g.101) and *Bipolaris orzyae* (B.o.177), were previously identified in the internal transcribed spacer (ITS) region [58] and were used in the present work. The cultivar name and fungal isolates are presented in Table 4. Isolate C.l.177 was identified as *Curvularia* sp. based on its colonial morphology, morphological characteristics, growth pattern, conidiophores and conidia.

#### 2.2.2. Molecular Identification through the Internal Transcribed Spacer (ITS) Region

The cultural characteristics of fungi isolates from Egypt were in agreement with those in previous studies [59,60]; thus, their identification was confirmed. However, the molecular characteristics were more precise and provided information on genetically pathogenic fungi. Accurate identification of pathogenic fungi is crucial in disease management of economically important crops, especially rice. A partial DNA sequence in the ITS region of the C.l.177 isolate was analyzed using the BLAST tool at the National Center of Biotechnology and Information site (http://www.ncbi.nlm.nih.gov). The partial DNA nucleotide sequence and analysis via BLAST and Genbank data showed the fungal isolate C.l.177 actually belonging to *C. lunata*. The homology of the *C. lunata* isolate to the Genbank strain reached 99%. The Genbank accession numbers of this isolate and other referenced isolates are shown in Table 5.

### 2.3. Antifungal Activity of Essential Oils

Table 6 shows that the EOs from the plants treated with AA and Trp at the concentrations of 100, 200 and 300 mg/L, and MLE at 2.5%, 5%, and 10%, compared with the control plants, were bioassayed against the growth of *F. graminearum*, *F. verticilliodies*, *B. oryzae* and *C. lunata*. The maximum fungal mycelial growth inhibition (FMGI) percentages of 94.57% and 92.63% against *F. verticilliodies* were found by MLE at 5% and 10%, followed by Trp (300 mg/L) and AA (100 mg/L) with FMGI values of 71.31% and 70.93%, respectively. In the case of *F. graminearum*, the best EO treatment that inhibited mycelial growth was Trp at the concentrations of 300 mg/L (FMGI 85.6%) and 200 mg/L (82.19%) compared with control (0%). Results of FMGI percentages against *B. oryzae* showed that the EOs obtained from the plants treated with MLE (10%), AA (300 mg/L) and AA (200 mg/L) had values of 84.46%, 82.19% and 80.68%, respectively. The EOs from plants treated with AA at the concentrations of 300 and 200 mg/L observed the highest FMGI against the growth of *C. lunata,* with values of 81.11% and 81.85%, respectively. Table 7 shows that the minimum inhibitory concentrations calculated for the essential oil treatments ranged between 250 and 400 mg/L, which were lower than those from Mancozeb as a chemically positive control of all fungal isolates (range from 20 to 40 mg/L).

Previous work showed that oil extracted with *n*-hexane at a concentration of 3% from *Pinus halepensis* showed an FMGI value of 80% against the growth of *B. oryzae*, while the EO from *Schinus terebinthifolius* observed the values of 74.44% and 71.66% at 3% and 2%, respectively [35]. Moreover, *Cymbopogon martinii*, *O. vulgare* and *Cinnamomum zeylanicum* EOs showed significant antifungal activities against *F. graminearum* and *F. culmorum* in wheat grains with FMGI percentages of 90.99% and 68.13%*,* respectively [61]. The methanol extract of *O. majorana* showed antifungal activity against some plant pathogenic fungi including *F. solani*, *Aspergillus niger* and *A. parasiticus* [62]. Moreover, (–)-terpinen-4-ol caused the mycelial growth inhibition of fungi *Aspergillus*, *Penicillium* and *Fusarium* species [63,64,65]. In addition, previous studies demonstrated that some monoterpenes preformed their antifungal action at the membrane level or membrane-embedded enzymes [66].

### 2.4. Efficacy of Seed Treatment with Essential Oils Against Seed-Borne Fungi

**Figure 1:**
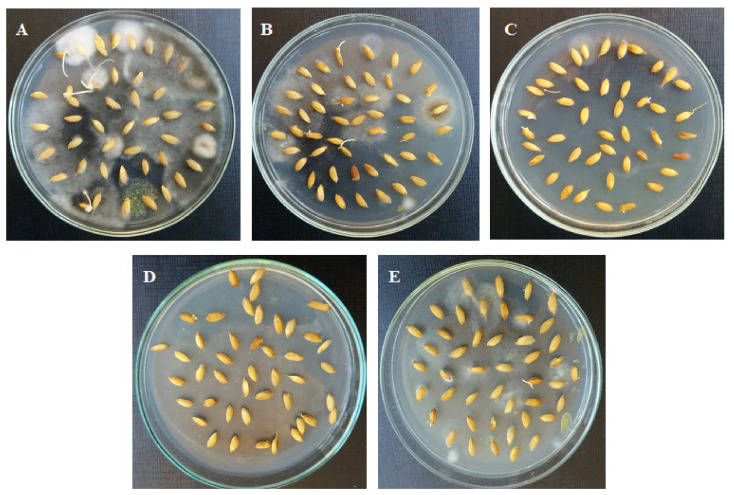
Antifungal activity of essential oils against rice seed-borne fungi of cultivar Giza177 in controlling seed-borne infection of *B. oryzae*, *F. verticilliodies*, *F. graminearum* and *C. lunata*; (**A**) control (untreated), and seeds treated with oil from (**B**) untreated plants, (**C**) plants treated with Trp, (**D**) with AA, and (**E**) with MLE.

From the previous antifungal activity bioassay results, the concentrations of the EOs from *O. majorana* plants treated with AA (300 mg/L), Trp (300 mg/L) and MLE at a concentration of 10% were used to evaluate their activity against seed-borne fungi as a seed treatment, and they were compared with the EO obtained from untreated plants and seeds without EOs. It is clear from the results of Table 8 and Figure 1 that the treatments with the EOs showed different levels of fungal inhibition compared with untreated grains. The percentage of infection in non-treated grains (naturally infected) was 82.5%, while the percentages of infection were suppressed as the rice seeds were treated with EOs extracted from *O. majorana* plants treated with Trp (300 mg/L), AA (300 mg/L) and MLE (10%), or from untreated plants, with values of 1.75%, 10.5%, 17.5% and 18.5%, respectively, compared to the control (82.5%).

The pathogens associated with discolored grains evidenced the transmission of pathogens through grains, which causes seedling and inflorescence abnormalities and rotting of grains in the soil [37,67,68,69]. Therefore, the present results showed that the EOs inhibited the fungi associated with rice seeds.

Finally, this study found that treatment of rice grains with EOs of *O. majorana* before sowing the rice in nurseries inhibited grain rot as well as enhanced grain germination and, thus, crop yield. EOs play important roles as antifungal agents and prevent the formation of fungal biofilms, the production of mycotoxins and the mechanisms of cellular communication [70].

## 3. Materials and Methods

### 3.1. Preparation of Aqueous Extracts of Moringa Oleifera, Ascorbic Acid and Tryptophan

Healthy leaves of *Moringa oleifera* Lam. were collected from the nursery of the Department of Floriculture, Ornamental Horticulture and Landscape Gardening, Faculty of Agriculture, Alexandria University, Egypt. The leaves were shade-dried and ground to a fine powder using an electric grinder. About 100 g of powdered leaves was soaked in 1000 mL of distilled water at room temperature for 24 h, then filtered through cotton plugs and then with filter paper (Whatman No. 1). The obtained moringa leaf extract (MLE) was prepared at the concentrations of 2.5%, 5% and 10%. Ascorbic acid (AA) and Tryptophan (Trp) were prepared at the concentrations of 100, 200 and 300 mg/L.

### 3.2. Field Study

The field study was carried out during two successive seasons of 2018 and 2019 at the Nursery of Floriculture, Department of Ornamental Horticulture and Landscape Gardening, Faculty of Agriculture, Alexandria University, Egypt. Sweet marjoram (*Origanum majorana* L.) seeds were sown in March and transplanted after one month in a mixture of sand and clay (1:1 *v/v*) for two seasons, 2018 and 2019, where four plants for each treatment were chosen. The soil mixture analysis was done according to Jackson [71] and illustrated in Table 9.

The plants were sprayed with MLE at different concentrations (2.5%, 5% and 10%) [72] as well as (AA) and (Trp) with concentrations of 100, 200 and 300 mg/L every 30 days starting from the 1st of May until the 1st of July in both seasons (the plants were sprayed three times: first of May, first of June and first of July) as shown in Table 10. The control plants were sprayed with tap water. The plants were harvested on the 1st of August in both seasons. In both seasons, all plants were fertilized with nitrogen-phosphorus-potassium (N-P-K) (Milagro Amino leaf 20-20-20) at a rate of 2 g/pot. Fertilization was repeated every 30 days throughout the growing season.

### 3.3. Vegetative Growth and Chemical Parameters

Vegetative growth parameters including plant height (cm), plant diameter (cm), number of branches per plant, leaf fresh weight (g), leaf dry weight (g) and leaf area (cm^2^) [73] as well as the chemical parameters were measured. Chlorophyll content of fresh leaves was determined as SPAD unites at the end of the season using Minolta (chlorophyll meter) SPAD 502. Carbohydrate content of the leaves was determined according to Dubois et al. [74].

### 3.4. Extraction of the Essential Oils

Essential oils (EOs) were extracted by a water distillation method using a Clevenger-type apparatus [75]. About 50 g from each *O. majorana* leaf from different treatments was put in a 2-L flask and hydrodistillated for 3 h. The collected essential oils were put in brown bottles and kept dry at 4 °C in the refrigerator. The amount of essential oils obtained from the treated plants, the oil percentage (%), was calculated according to the following formula:

$$Oil\%=\frac{A}{B}\times 100$$

where *A* is the quantity of the EO from plants (mL), and *B* is the weight of the plant sample (g).

### 3.5. GC/MS Analysis of the Essential Oils

The obtained EOs from *O. majorana* leaves were analyzed for their chemical compositions using a Trace GC 1300-TSQ Quantum mass spectrometer (Thermo Scientific, Austin, TX, USA) with a direct capillary column TG–5MS (30 m × 0.25 mm × 0.25 μm film thickness). The column oven temperature was initially held at 60 °C and then increased by 5 °C/min to 250 °C, held for 2 min, and then increased to 300 °C at 5 °C/min. The injector and MS transfer line temperatures were maintained at 270 °C. Helium was used as the carrier gas at a constant flow rate of 1 mL/min. The solvent delay was 3 min, and diluted samples of 1 μL were injected automatically using an Autosampler AS3000 (Thermo Scientific, Austin, TX, USA) coupled with GC in the split mode. EI mass spectra were collected at 70 eV ionization voltages over the range of *m*/*z* 40–650 in full scan mode. The ion source and transfer line were set at 200 °C and 280 °C, respectively. The components were identified by comparison of their retention times and mass spectra with those of the WILEY 09 and NIST 14 mass spectral database. GC/MS with Xcalibur 3.0 data system has threshold values to confirm that all the MS of the compounds were attached to the library. Therefore, measuring their standard index (SI) and reverse standard index (RSI), values ≥ 650 are acceptable to confirm the compounds [35,75,76,77,78].

### 3.6. In Vitro Study for Fungal Isolation

The experiments were carried out at the Department of seed pathology, Plant Pathology Research Institute, Agriculture Research Center (ARC), Egypt. Rice variety Sakha 101 was taken from the Rice Research and Training Center (RRTC), Sakha, Kafr El-Sheikh. Randomly, 100 grains were picked from the Giza 177 cultivar, which was obtained according to the International Rules of Seed Testing Association [79]. These grains were disinfected, placed in Petri dishes on potato dextrose agar media (PDA) and incubated at 25 °C in the dark for 4–5 days. Isolation and purification techniques were done following the methods of Mothlagh and Kavian [80]. Three fungal isolates, *Fusarium verticilliodies* (F.v.101), *F. graminearum* (F.g.101) and *Bipolaris orzyae* (B.o.177), which were previously isolated and identified from Sakha 101 and Giza 177 rice cultivars, were used in the present study [58].

### 3.7. In Vitro Study for Fungal Identification

#### 3.7.1. Culture Characteristics and Microscopic Examination

Basic culture characteristics, such as color and growth pattern, were studied on the PDA medium. Fungal growth was stained with lactin-blue phenol and examined under microscopic light for fungal diagnostic properties. Innate isolation was defined on the basis of the description by Sivanesan [59].

#### 3.7.2. Molecular Identification through the Internal Transcribed Spacer (ITS) Region

Total genomic DNA was extracted from the fungal growth according to Edel et al. [81]. The DNA pellet was dissolved in 100 µL of TE buffer and stored at 4 °C until use. The internal transcribed spacer (ITS) region was amplified using two universal primers: ITS1 (5′-TCCGTAGGTGAACCTGCGG-3′) and ITS4 (5′-TCCTCCGCTTATTGATATGC-3′). PCR amplification was carried out in a total volume of 25 μL containing 2 μL of template DNA, 10 mM Tris-HCl (pH 8.8), 50 mM KCl, 1.5 mM MgCl_2_, 0.2 mM of each dNTP, 10 pmol of each primer and 0.5 unit Dynazyme TM II DNA Polymerase. PCR amplification was performed as one cycle at 95 °C for 1 min followed by 35 cycles each with 30 s at 94 °C for denaturation, 2 min at 55 °C for annealing and 1 min at 72 °C for elongation. Reactions were then incubated at 72 °C for 10 min for the final extension. PCR products were separated on a 1.5% agarose gel in 1X TAE buffer at 120 Volt, stained with EtBr and visualized under UV light [82].

#### 3.7.3. Sequencing of the Amplified ITS Region

The amplified ITS1-5.8s and ITS2 regions were sent for sequencing. The products were sequenced by the use of a Big Dye terminator cycle sequencing kit and resolved on an ABI PRISM model 310 automated DNA sequencer from Sigma Company. Identification was confirmed by applying a BLAST search on the National Center for Biotechnology information (NCBI) site (http://www.ncbi.nlm.nih.gov) using the obtained sequences of the amplified regions. The ITS1-5.8s and ITS2 region sequences have been deposited in the Genbank database.

### 3.8. Antifungal Activity and Minimum Inhibitory Concentration (MIC) Assays of Essential Oils (First Investigation)

The EOs were prepared at a concentration of 2000 mg/L by diluting the oil in 10% dimethyl sulfoxide (DMSO) with the addition of a few drops of Tween-80 [83]. The antifungal activity of the EOs from *O. majorana* leaves was assessed against four grain-borne fungi (*F. verticilliodies, F. graminearum*, *B. orzyae* and *C. lunata*) by radial growth. The isolates were cultivated on PDA medium. Then, a single 6-mm culture disk was taken from actively growing cultures using a cork-borer and placed in the middle of the Petri dishes. Sterilized filter discs of 17 mm diameter loaded with 50 μL of 2000 mg/L of each oil were placed around the fungal disk and compared with referenced fungicide drug Mancozeb at 200 mg/L. The plates were incubated at 28 °C for 5 days, and three replications were used for each fungal pathogen [84,85]. Fungal mycelial growth inhibition (%) of the tested fungi was calculated with the following formula:

$$Mycelial\;growth\;inhibition\;\left(\%\right)=\frac{G0-Gt}{G0}\times 100$$

where *G*0 and *G*t are the average diameters (mm) of fungal colonies under the control and experimental treatments, respectively. The minimum inhibitory concentrations (MICs) of the essential oils prepared at concentrations of 200–2000 mg/L and Mancozeb (reference chemical fungicide) prepared at concentrations of 10–200 mg/L were assessed using the broth dilution method according to CLSI [86].

### 3.9. Efficacy of Seed Treatment with Selected Essential Oils Against Seed-Borne Fungi (Second Investigation)

The effectiveness of essential oils against seed-borne fungi was evaluated on 400 rice grains by treating them with oils from the most effective concentrated treatment for the plants based on in vitro testing, along with untreated control (sterile water), and then placing them on PDA Petri dishes followed by incubation for 5 days at 28 °C [87,88]. The percentage rice grain infection was calculated by the following formula [89]:

$$Rice\;grain\;infection\;\left(\%\right)=\frac{No.\;of\;seed\;containing\;a\;particular\;fungus}{Total\;seeds}\times 100$$

### 3.10. Statistical Analysis

The experiment was conducted in a complete randomized block design (RCBD) containing 10 treatments with three replicates. The collected data of vegetative growth and chemical parameters from both seasons were subjected to one-way analysis of variance (ANOVA) using the SAS program, SAS Institute [90]. Data on mycelial growth inhibition (%) as affected by essential oils from plants treated with AA and tryptophan at concentrations of 100, 200 and 300 mg/L and *M. oleifera* leaf extract (2.5%, 5% and 10%) were subjected to one-way ANOVA. The means of the individual factors and their interactions were compared by the least significant difference (L.S.D) test at 5% level of probability according to Snedecor and Cochran [91].

## 4. Conclusions

This study found that different foliar applications of ascorbic acid, tryptophan and moringa leaf extract on *O. majorana* plants significantly affected vegetative growth and chemical constituents compared with untreated plants. Essential oils had potential antifungal activity against the growth of *F. verticilliodies*, *F. graminearum*, *B. oryzae* and *C. lunata.* Furthermore, the most effective essential oils were obtained from the *O. majorana* plants treated with ascorbic acid and tryptophan at 300 mg/L, whereas moringa leaf extract is suggested to be used at 10% to inhibit the growth of these fungi when applied to seed rice grains before sowing. Even though this is a high amount, the raw material of moringa is readily available as it is a fast-growing tree. This study suggests using these essential oils as a biofungicide against seed-born fungi, especially in treatments before sowing.

## Figures and Tables

**Table 1:** Vegetative parameters of *O. majorana* plants treated with ascorbic acid (AA), tryptophan (Trp) and moringa leaf extract (MLE) in two successive seasons.

Treatments	Plant Height (cm)		Plant Diameter (cm)		Branch Number		Leaf Fresh Weight (g)		Leaf Dry Weight (g)		Leaf Area	
	2018	2019	2018	2019	2018	2019	2018	2019	2018	2019	2018	2019
Control	17.16 ^d*^ ± 1.87	23.16 **^b^** ± 3.95	21.33 **^b^** ± 0.76	26.00 **^e^** ± 1.39	95.16 **^b^** ± 8.54	143.50 **^c^** ± 14.23	29.62 **^e^** ± 1.92	51.86 **^c^** ± 0.23	11.55 **^b^** ± 4.29	12.42 **^d^** ± 1.01	0.323 **^f^** ± 0.030	0.453 **^d^** ± 0.063
AA 100 mg/L	20.00 **^ab^** ± 1.39	25.66 **^ab^** ± 2.46	23.58 **^ab^** ± 0.76	29.50 **^ab^** ± 0.25	150.00 **^a^** ± 15.78	223.00 **^a^** ± 13.61	35.77 **^abc^** ± 1.45	60.68 **^ab^** ± 6.73	19.11 **^ab^** ± 1.82	21.20 **^abc^** ± 4.83	0.431 **^becd^** ± 0.114	0.713 **^ab^** ± 0.135
AA 200 mg/L	20.66 **^ab^** ± 3.12	27.08 **^a^** ± 3.95	23.66 **^ab^** ± 2.75	30.41 **^ab^** ± 0.52	150.33 **^a^** ± 8.69	230.16 **^a^** ± 59.25	37.56 **^ab^** ± 6.29	64.12 **^a^** ± 2.02	19.41 **^ab^** ± 6.74	21.57 **^ab^** ± 7.14	0.365 **^ef^** ± 0.012	0.412 **^d^** ± 0.034
AA 300 mg/L	21.33 **^a^** ± 2.24	27.08 **^a^** ± 3.16	24.75 **^a^** ± 0.66	31.08 **^a^** ± 0.62	152.33 **^a^** ± 9.50	232.16 **^a^** ± 10.39	39.08 **^a^** ± 6.29	64.37 **^a^** ± 2.68	20.72 **^a^** ± 5.62	25.03 **^a^** ± 4.79	0.733 **^a^** ± 0.053	0.852 **^a^** ± 0.07
Trp 100 mg/L	19.58 **^abc^** ± 4.15	25.33 **^ab^** ± 2.50	22.50 **^ab^** ± 1.56	27.33 **^cde^** ± 0.38	139.83 **^a^** ± 6.00	214.50 **^ab^** ± 36.87	30.24 **^de^** ± 1.34	54.20 **^bc^** ± 3.25	11.97 **^b^** ± 4.88	13.42 **^cd^** ± 1.31	0.392 **^cedf^** ± 0.025	0.458 **^cd^** ± 0.051
Trp 200 mg/L	19.75 **^abc^** ± 0.66	25.50 ^a**b**^ ± 3.27	22.58 **^ab^** ± 0.52	28.66**^bcd^** ± 0.2	143.00 **^a^** ± 7.54	219.00 **^a^** ± 38.50	30.61 **^cde^** ± 1.88	54.96 **^bc^** ± 8.56	12.48 **^b^** ± 0.86	14.90 **^bcd^** ± 5.47	0.517 **^b^** ± 0.120	0.611 **^bc^** ± 0.084
Trp 300 mg/L	19.75 **^abc^** ± 2.17	25.50 **^ab^** ± 1.80	23.25 **^ab^** ± 4.02	28.83 **^bc^** ± 1.84	145.16 **^a^** ± 4.53	221.50 **^a^** ± 21.21	31.12 **^cde^** ± 3.00	57.09 **^abc^** ± 3.18	13.41 **^ab^** ± 2.55	15.56 **^bcd^** ± 1.06	0.474 **^cbd^** ± 0.036	0.61 **^bc^** ± 0.045
MLE 2.5%	17.91 **^cd^** ± 1.75	24.33 **^b^** ± 2.02	21.91 **^b^** ± 1.28	26.66 **^de^** ± 3.35	107.16 **^b^** ± 26.27	168.16 **^bc^** ± 13.32	32.54 **^bcde^** ± 0.85	58.08 **^abc^** ± 2.26	13.47 **^ab^** ± 7.26	16.93 **^bcd^** ± 5.50	0.385 **^def^** ± 0.006	0.477 **^cd^** ± 0.077
MLE 5%	18.75 **^bcd^** ± 2.61	24.50 **^ab^** ± 2.00	22.41 **^ab^** ± 1.18	26.75 **^de^** ± 1.14	136.83 **^a^** ± 17.32	209.16 **^ab^** ± 20.93	33.77 **^abcde^** ± 1.98	58.66 **^abc^** ± 5.03	15.46 **^ab^** ± 1.28	17.22 **^abcd^** ± 7.69	0.5 ^b**c**^ ± 0.080	0.741 **^ab^** ± 0.035
MLE 10%	19.66 **^abc^** ± 1.25	24.91 **^ab^** ± 3.18	22.41 **^ab^** ± 0.80	27.08 **^cde^** ± 0.52	138.16 **^a^** ± 31.60	213.66 **^ab^** ± 14.15	35.37 **^abcd^** ± 0.27	59.06 **^abc^** ± 3.37	16.39 **^ab^** ± 5.97	20.09 **^abcd^** ± 4.27	0.497 **^bc^** ± 0.0155	0.669 **^b^** ± 0.190

* No significance between means have the same letters (^a–e^) within the same column.

**Table 2:** Chemical parameters of *O. majorana* plants treated with AA, Trp and MLE in two successive seasons.

Treatment	Total Chlorophyll Content (SPAD)		Total Carbohydrate Content (%)		Oil Percentage (%)	
	2018	2019	2018	2019	2018	2019
Control	44.68 ^c*^ ± 10.10	47.66 ^c^ ± 10.80	7.50 ^f^ ± 0.45	7.66 ^g^ ± 0.47	0.664 ^b^ ± 0.050	0.658 ^abc^ ± 0.219
AA 100 mg/L	53.13 ^abc^ ± 3.07	54.05 ^bc^ ± 4.29	8.71 ^c^ ± 0.46	8.86 ^d^ ± 0.47	0.892 ^ab^ ± 0.168	0.535 ^c^ ± 0.128
AA 200 mg/L	57.01 ^abc^ ± 11.93	58.10 ^abc^ ± 5.96	8.81 ^c^ ± 0.45	9.04 ^c^ ± 0.44	0.864 ^ab^ ± 0.094	0.546 ^bc^ ± 0.178
AA 300 mg/L	57.71 ^ab^ ± 2.86	58.08 ^abc^ ± 3.28	8.86 ^c^ ± 0.47	9.08 ^c^ ± 0.45	0.982 ^a^ ± 0.462	0.737 ^abc^ ± 0.240
Trp 100 mg/L	45.33 ^c^ ± 14.07	51.31 ^bc^ ± 8.58	8.01 ^e^ ± 0.45	8.42 ^e^ ± 0.45	0.726 ^ab^ ± 0.199	0.683 ^abc^ ± 0.148
Trp 200 mg/L	47.06 ^bc^ ± 3.79	53.53 ^bc^ ± 2.91	8.06 ^e^ ± 0.45	7.98 ^f^ ± 0.45	0.8 ^ab^ ± 0.156	0.826 ^a^ ± 0.245
Trp 300 mg/L	52.55 ^abc^ ± 1.16	53.90 ^bc^ ± 3.51	8.37 ^d^ ± 0.44	8.44 ^e^ ± 0.40	0.748 ^ab^ ± 0.054	0.822 ^ab^ ± 0.148
MLE 2.5%	56.03 ^abc^ ± 5.04	60.11 ^ab^ ± 4.23	9.10 ^b^ ± 0.45	9.10 ^c^ ± 0.45	0.977 ^ab^ ± 0.218	0.799 ^abc^ ± 0.017
MLE 5%	56.28 ^abc^ ± 8.14	60.68 ^ab^ ± 11.48	9.73 ^a^ ± 0.15	9.29 ^b^ ± 0.45	0.917 ^ab^ ± 0.127	0.81 ^abc^ ± 0.146
MLE 10%	59.90 ^a^ ± 7.20	66.36 ^a^ ± 8.31	9.62 ^a^ ± 0.30	9.79 ^a^ ± 0.15	1.065 ^a^ ± 0.313	0.867 ^a^ ± 0.058

* No significance between means have the same letters (^a–f^) within the same column.

**Table 3:** Chemical compositions of the essential oils obtained from plants treated with AA, Trp and MLE.

	Percentage in the Oil (%)									
Compound Name	Control (Untreated Plants)	AA (mg/L)			MLE (%)			Trp (mg/L)		
		100	200	300	2.5	5	10	100	200	300
*α*-Pinene	0.53 (913,910) *	0.41 (937,921)	0.61 (916,910)	ND	0.68 (914,911)	ND	ND	ND	ND	0.45 (920,914)
Sabinene	6.83 (863,861)	8.66 (878,876)	7.82 (877,876)	5.49 (918,909)	6.89 (917,911)	2.76 (937,930)	3.13 (932,924)	5.79 (918,917)	3.53 (929,928)	6.67 (935,929)
*α*-Terpinene	0.42 (927,923)	ND	0.84 (908,907)	0.33 (923,919)	3.55 (909,907)	ND	0.8 (923,919)	0.7 (921,905)	ND	ND
2-Carene	ND	2.9 (881,881)	1.42 (886,885)	0.73 (869,864)	ND	ND	0.61 (868,867)	ND	ND	ND
Cymene	4.5 (865,850)	3.83 (846,809)	4.55 (864,846)	3.29 (829,813)	3.9 (906,888)	3.53 (914,903)	3.04 (929,909)	4.41 (824,815)	7.2 (893,875)	17.75 (896,884)
*γ*-Terpinene	1.56 (930,925)	4.88 (898,869)	3.17 (901,875)	2.53 (934,928)	7.65 (887,885)	0.96 (938,930)	2.84 (931,926)	2.53 (931,926)	0.67 (938,930)	0.32 (881,877)
4-Thujanol (Sabinene hydrate)	10.51 (885,884)	11.3 (865,865)	10.38 (875,874)	12.18 (866,862)	24.47 (867,865)	46.47 (847,845)	33.28 (873,871)	37.17 (911,908)	39.88 (865,862)	9.13 (925,915)
Terpineol (*cis-β*-(1-Terpinenol))	33.17 (814,812)	26.76 (856,839)	28.44 (803,787)	38.06 (820,806)	ND	ND	ND	ND	ND	ND
*cis*-*p*-2-Menthen-1-ol	1.29 (874,871)	1.2 (874,870)	1.4 (877,873)	1.09 (876,875)	1.29 (892,887)	1.39 (887,835)	2 (902,896)	1.68 (882,878)	1.68 (878,872)	ND
Terpinen-4-ol	22.25 (813,813)	21.58 (815,815)	21.73 (824,824)	17.42 (837,837)	33.34 (815,815)	27.65 (870,864)	32.04 (829,829)	26.09 (822,822)	25.9 (819,818)	38.35 (902,896)
*cis*-Piperitol	0.23 (847,800)	0.19 (845,806)	0.22 (856,814)	0.12 (839,790)	0.18 (884,841)	0.18 (920,893)	0.27 (897,877)	0.21 (870,829)	0.27 (871,811)	1.82 (858,831)
*α*-Terpineol	7.78 (824,765)	6.24 (861,827)	7.37 (860,814)	7.18 (847,815)	8.88 (857,857)	9.48 (884,883)	12.58 (842,832)	10.67 (821,772)	9.22 (873,873)	7.91 (933,921)
Linalyl acetate	2.33 (846,845)	ND	2.41 (819,817)	2.45 (822,821)	0.76 (907,903)	1.86 (933,930)	1.01 (926,923)	0.92 (897,895)	3.72 (862,858)	ND
*cis*-Sabinene hydrate acetate	2.24 (852,811)	5.06 (867,780)	2.09 (870,825)	1.91 (885,786)	3.64 (888,789)	0.33 (925,818)	1.7 (913,811)	2.88 (922,877)	2.03 (888,790)	0.86 (922,859)
1-Terpinen-4-yl acetate	0.35 (838,830)	0.48 (846,838)	0.54 (822,814)	0.32 (906,862)	0.34 (842,833)	0.27 (867,855)	0.67 (836,828)	0.41 (850,841)	0.55 (838,829)	ND
2,5-Dimethyl-3-hexyne-2,5-diol	0.62 (796,757)	0.32 (810,766)	0.53 (794,758)	0.19 (807,757)	0.21 (822,780)	0.36 (823,786)	0.15 (819,771)	0.53 (822,782)	0.36 (817,779)	ND
Caryophyllene	2.26 (894,850)	2.71 (812,810)	3.19 (865,806)	2.71 (812,809)	2.1 (899,870)	1.84 (918,918)	2.02 (913,903)	2.01 (891,862)	1.96 (890,866)	1.46 (915,913)
*γ*-Elemene	0.61 (863,860)	1.16 (840,839)	0.96 (837,835)	1.36 (840,839)	0.82 (857,855)	0.53 (869,865)	0.91 (846,844)	0.83 (860,858)	0.42 (878,874)	ND
(-)-Spathulenol	0.72 (863,862)	0.7 (888,873)	0.75 (859,859)	0.67 (900,882)	0.23 (885,883)	0.75 (921,906)	0.61 (915,902)	0.88 (865,864)	0.84 (906,889)	4.52 (901,900)
Caryophyllene oxide	0.22 (886,884)	0.19 (884,882)	0.21 (904,903)	0.29 (884,881)	0.09 (916,912)	0.43 (935,933)	0.33 (922,921)	0.16 (926,924)	0.41 (916,915)	0.29 (776,762)
Monoterpenes (%)	94.61	93.81	93.52	93.29	95.78	95.24	94.12	93.99	95.01	83.26
Sesquiterpenes (%)	3.81	4.76	5.11	5.03	3.24	3.55	3.87	3.88	3.63	6.27
Non- oxygenated constituents (%)	16.71	24.55	22.56	16.44	25.59	9.62	13.35	16.27	13.78	26.65
Oxygenated constituents (%)	81.71	74.02	76.07	81.88	73.43	89.17	84.64	81.60	84.86	62.88

* Values in parentheses are (SI: Standard Index, RSI: Reverse Standard Index); ND: Not detected.

**Table 4:** Correction coefficient (r) among the treatments affecting the monoterpenoids and sesquiterpenoids or oxygenated and non-oxygenated constituents of the essential oils.

	Control	AA 100 mg/L	AA 200 mg/L	AA 300 mg/L	MLE 2.5%	MLE 5%	MLE 10%	Trp 100 mg/L	Trp 200 mg/L	Trp 300 mg/L
Control	1									
AA 100 mg/L	0.99	1								
AA 200 mg/L	0.997	0.999	1							
AA 300 mg/L	1.000	0.991	0.995	1						
MLE 2.5%	0.989	0.999	0.997	0.985	1					
MLE 5%	0.996	0.980	0.987	0.998	0.972	1				
MLE 10%	0.999	0.987	0.993	1.000	0.980	0.999	1			
Trp 100 mg/L	1.000	0.993	0.997	1.000	0.988	0.997	0.999	1		
Trp 200 mg/L	0.999	0.989	0.994	1.000	0.983	0.999	1.000	1.000	1	
Trp 300 mg/L	0.963	0.985	0.977	0.955	0.990	0.937	0.949	0.962	0.954	1

**Table 5:** Fungal isolates obtained from discolored rice grains and Genbank accession number codes.

Isolate Code	Cultivar	Isolated Fungi	Accession Number	Reference
F.g.101	Sakha 101	*Fusarium graminearum*	MK450469	[58]
F.v.101		*Fusarium verticilliodies*	MK450470	[58]
B.o.177	Giza 177	*Bipolaris oryzae*	MK450473	[58]
C.l.177		*Curvularia lunata*	MK450466	(This study)

**Table 6:** Antifungal activity of the essential oils against the growth of *F. verticilliodies*, *F. graminearum*, *B. oryzae* and *C. lunata*.

Oil from Plant Treated with	Inhibition Percentage %			
	*F. verticilliodies*	*F. graminearum*	*B. oryzae*	*C. lunata*
Control (without oil)	0.00 ***^f^*** ± 0.00 *	0.00 ***^e^*** ± 0.00	0.00 ***^f^*** ± 0.00	0.00 ***^e^*** ± 0.00
Control (oil from untreated plants)	63.56 ***^e^*** ± 0.77	62.87 ***^d^*** ± 0.37	45.83 ***^e^*** ± 1.65	50.74 ***^d^*** ± 0.74
AA (100 mg/L)	70.93 ***^cd^*** ± 0.67	70.07 ***^b^*** ± 1.00	77.65 ***^bcd^*** ± 1.65	74.07 ***^b^*** ± 0.97
AA (200 mg/L)	69.76 ***^b^*** ± 0.67	70.07 ***^b^*** ± 1.00	80.68 ***^abc^*** ± 1.73	81.85 ***^a^*** ± 0.97
AA (300 mg/L)	69.76 ***^bc^*** ± 0.67	68.18 ***^bc^*** ± 0.65	82.19 ***^ab^*** ± 3.23	81.11 ***^a^*** ± 0.64
Trp (100 mg/L)	65.50 ***^de^*** ± 1.39	67.80 ***^cd^*** ± 4.60	75.37 ***^d^*** ± 1.00	70.74 ***^c^*** ± 0.97
Trp (200 mg/L)	67.82 ***^cd^*** ± 1.02	82.19 ***^a^*** ± 2.10	77.65 ***^bcd^*** ± 1.36	72.59 ***^bc^*** ± 1.33
Trp (300 mg/L)	71.31 ***^b^*** ± 0.38	85.60 ***^a^*** ± 0.75	79.54 ***^abcd^*** ± 0.65	74.81 ***^b^*** ± 1.95
MLE (2.5%)	65.11 ***^de^*** ± 1.34	63.25 ***^cd^*** ± 2.00	76.13 ***^cd^*** ± 0.65	73.70 ***^bc^*** ± 0.74
MLE (5%)	94.57 ***^a^*** ± 1.39	70.07 ***^b^*** ± 0.37	79.54 ***^abcd^*** ± 0.65	73.70 ***^bc^*** ± 0.97
MLE (10%)	92.63 ***^a^*** ± 0.38	71.96 ***^b^*** ± 0.37	84.46 ***^a^*** ± 3.10	75.18 ***^b^*** ± 1.33
Mancozeb (200 mg/L) **	53.5 ± 2.45	65.15 ± 2.62	77.35 ± 2.54	63.33 ± 3.33

*: Means with the same letters (^a–f^) in the same column are not significantly different according to LSD 0.05; ** Reference fungicide.

**Table 7:** Minimum inhibitory concentrations (MICs) of the essential oil treatments and reference fungicide.

Oil from Plant Treated with	Minimum Inhibitory Concentration (MIC mg/L)			
	*F. verticilliodies*	*F. graminearum*	*B. oryzae*	*C. lunata*
Control (Oil from untreated plants)	300	300	400	400
AA (100 mg/L)	350	350	250	350
AA (200 mg/L)	350	350	250	250
AA (300 mg/L)	350	350	250	250
Trp (100 mg/L)	300	350	350	350
Trp (200 mg/L)	350	250	250	350
Trp (300 mg/L)	350	250	250	350
MLE (2.5%)	300	300	250	350
MLE (5%)	200	350	250	350
MLE (10%)	200	350	250	350
Mancozeb(reference fungicide)	40	30	20	25

**Table 8:** Effect of treatments with selected essential oils of *O. majorana* on seed-born fungi.

Fungi	Seeds without Oil	Oils from Plants Treated with			
		Untreated Plants	Ascorbic Acid (300 mg/L)	Moringa Extract (10%)	Tryptophan (300 mg/L)
*F. verticilliodies*	28	24	0.0	0.0	0.0
*F. graminearum*	21	18	42	0.0	0.0
*B. oryzae*	232	32	0.0	70	7.0
*C. lunata*	49	0.0	0.0	0.0	0.0
Total grains					
Healthy grains	70	326	358	330	393
Infected grains	330	74	42	70	7
Rice grain infection (%)	82.5	18.5	10.5	17.5	1.75

**Table 9:** Some chemical analyses of the used mixture soil for two successive seasons, 2018 and 2019.

Season	pH	EC (dSm^−1^)	Soluble Cations (meq/L)				Soluble Anions (meq/L)		
			Ca^++^	Mg^++^	Na^+^	K^+^	HCO_3_^−^	Cl^−^	SO_2_^−−^
2018	8.08	2.53	18.20	14.20	23.91	4.49	7.20	21.00	27.10
2019	8.13	2.03	13.22	3.0	16.21	3.79	6.52	16.93	25.21

**Table 10:** Different treatments used and their concentrations.

Treatment	Concentration	Treatment	Concentration
Control plants	Tap water	Trp	200 mg/L
AA (ascorbic acid)	100 mg/L	Trp	300 mg/L
AA	200 mg/L	(moringa leaf extract) MLE	2.5%
AA	300 mg/L	MLE	5%
Trp (tryptophan)	100 mg/L	MLE	10%
